# Arterial stiffness is a predictor for acute kidney injury following coronary artery bypass graft surgery

**DOI:** 10.1186/s13019-019-0873-3

**Published:** 2019-03-07

**Authors:** Sharlene A. Greenwood, Emmanuel Mangahis, Ellen M. Castle, Joe Wang, Jackie Campbell, Ranjit Deshpande, Satish Jayawardene

**Affiliations:** 1 Department of Therapies, King’s College Hospital, London, UK; 2 Department of Renal Medicine, King’s College Hospital, London, UK; 3 Renal Sciences, Department of Transplantation, Immunology and Mucosal Biology, King’s College London, London, UK; 4 Department of Renal Medicine, Epsom & St Helier Hospital, London, UK; 5 Faculty of Health and Social Care, University of Northampton, Northampton, UK; 6 Department of Cardiovascular Sciences, King’s College Hospital, London, UK

**Keywords:** Arterial stiffness, Pulse wave velocity, Acute kidney injury, Coronary artery bypass graft surgery

## Abstract

**Background:**

Cardiac surgery-associated acute kidney injury (CSA-AKI) is a serious postoperative complication of cardiac surgery, an episode of which impacts on patient morbidity and mortality. Pulse wave velocity (PWV; a non-invasive measurement tool to assess arterial stiffness) has been shown to predict kidney disease progression, and cardiovascular and all-cause mortality in patients with chronic kidney disease. We hypothesised that PWV would also predict acute kidney injury in subjects who have undergone non-valve repair elective coronary artery bypass graft (CABG) surgery .

**Methods:**

This was a prospective, observational, exploratory study. PWV was determined with a Vicorder device, together with standard clinical and biochemical parameters. AKI staging was defined according to the Kidney Disease Improving Global Outcomes (KDIGO) Clinical Practice Guidelines.

**Results:**

137 patients were included in the study. 85% were male, and mean age was 66.3 years (SD = 9.7 years). There were 40 episodes (29%) of CSA-AKI. Each 1 unit increase in PWV score was associated with a 1.5 fold greater odds of a CSA-AKI event (*p* = 0.006(odds ratio = 1.5; confidence interval:1.13–2.10). A 1 unit increase in estimated glomerular filtration rate resulted in an estimated 85% decrease in the odds of developing AKI, each year, men have an odds reduction of 15% of developing AKI compared with females and each 1 year increase in age lowered the odds of developing AKI by 87%.

**Conclusions:**

This pilot exploratory study revealed that PWV, assessed prior to non-valve repair elective CABG surgery, independently predicts CSA-AKI events. PWV is a simple, non-invasive technique that could potentially be used to risk stratify for CSA- AKI following elective cardiac surgery.

**Trial registration:**

ClinTrial.Gov NCT02364427. Registered 18 February 2015.

## Background

Acute Kidney Injury (AKI) affects around 20% of all hospitalised patients [1] and has both major long-term health and socioeconomic implications [2, 3]. Longitudinal studies have demonstrated that an episode of AKI can significantly increase both the risk of development of chronic kidney disease and early mortality [4]. Published data that considers non-valve repair coronary artery bypass graft (CABG) patients report an incidence rate of AKI to be between 2 and 12% [5, 6]. Our research team performed a retrospective analysis of incidence of AKI in patients undergoing elective, isolated CABG surgery at our hospital from January to December 2012. Of a total of 219 patients, 42 patients were classified as having post-operative AKI according to serum AKI criteria (unpublished data). This represents an incidence of 19.6%.

Currently there is no specific therapy for AKI and the management of patients is completely supportive. Furthermore, in the absence of more sensitive biomarkers, it would appear that even a non-severe episode of AKI already confers a worse prognosis with regards to the risk of development of CKD and long term survival in patients. Thus perhaps focus should be on prevention, and early detection of risk, of AKI as opposed to cure. This is even more pertinent when one considers that the National Confidential Enquiry into Patient Outcomes and Death (NCEPOD) report in 2009 [1] identified 30% of cases of AKI as being ‘preventable’ and a deficiency of care in 50% of cases. Thus ‘preventable’ cases of AKI could potentially save £130–186 million per annum [3].

Although factors such as diabetes, older age and low eGFR are all associated with increased risk of AKI, there is no standardized scoring method for risk [7]. In addition, it is not fully clear why some patients suffer AKI while others, with similar co-morbidities, do not. Arterial stiffness (AS) can be assessed non-invasively by measurement of pulse wave velocity (PWV) [8]. Kidney function is highly reliant on renal perfusion and good vascular health. This relationship is emphasised by studies that show renal functional decline is associated with an increase in risk of adverse cardiovascular events and death [9]. This has led to a focus on ‘non-traditional’ risk factors in the renal population such as AS and left ventricular hypertrophy (LVH). Increased AS has been shown to be be an independent risk factor for cardiovascular events in ESRF and in renal transplant recipients [10, 11].

The aim of this exploratory study was to investigate whether arterial stiffness, as defined by measuring Pulse Wave Velocity, a simple non-invasive measurement score, could be identified as an independent risk factor for development of post-coronary artery bypass graft acute kidney injury.

## Methods

### Participants

Participants were approached during routine surgical pre-assessment clinic at King’s College Hospital (KCH) in London. They were included if they were aged > 18 years, due to have CABG surgery alone with no concomitant valve surgery, and were able to give written consent. Patients were excluded if they had aortic grafts or renal stents, had renal function reflecting CKD stage 4 or 5, were receiving dialysis treatment, or had a significant psychiatric illness (including anxiety, mood and untreated eating disorders).

### Study procedure

Patients were assessed in a quiet treatment room in the pre-assessment clinic in the early afternoon. Data collection included blood and urine samples, patient demographics and anthropometrics (height, weight, and waist circumference), followed by blood pressure (BP), heart rate (HR), and PWV. All outcome assessments were performed at the same study visit.

### Primary outcome

Pulse Wave Velocity (PWV), a measurement of arterial stiffness, was assessed at the systemic region (carotid-femoral PWV), which is the gold standard method [12], using the Vicorder system (Skidmore Industries, UK). Conditions for assessment, as stated expert consensus [12], were adhered to for all measurements. The measurement protocol by Hickson et al. [13] was used, mathematically removing the additional femoral segment from the Vicorder standard protocol, to correct for any inherent bias at high arterial PWV. The average of 3 measurements (of 20 consecutive signals) was recorded at each time point.

### Acute kidney injury

The development of AKI was determined according to the Kidney Disease Improving Global Outcomes (KDIGO) Clinical Practice Guideline for Acute Kidney Injury criteria [14]. In brief, the guideline covers definition and staging of AKI based on the Risk, Injury, Failure; Loss, End-Stage Renal Disease (RIFLE) and Acute Kidney Injury Network (AKIN) criteria and studies on risk relationships.

### Blood tests

Venous blood was collected at the study visit. Serum creatinine (traceable to a calibrator tested with a reference method (IDMS – isotope dilution mass spectrometry) was analysed in the biochemistry department at King’s College Hospital.

### Resting BP and HR

After sitting quietly for 5 min, resting BP and HR were recorded in triplicate, with a 1-min interval between measurements, using an automated sphygmomanometer (Tango; SunTech Medical, Oxfordshire, UK). The average of 3 readings was recorded.

### Statistical analyses

Parameters are expressed as means ± SD (for normally distributed data) and median and interquartile range for non-parametric data. Differences between groups were assessed by the independent samples median test, t-test, or chi-squared test as appropriate to the data type. Correlations were assessed by the Pearsons correlation coefficient. Linear and logistic regression analyses were used to generate regression beta coefficients (B) and odds ratios for a range of baseline variables (age at assessment, gender, diabetes, eGFR) to determine factors that independently predicted the risk of a post-operative AKI event.

## Results

**Fig. 1:**
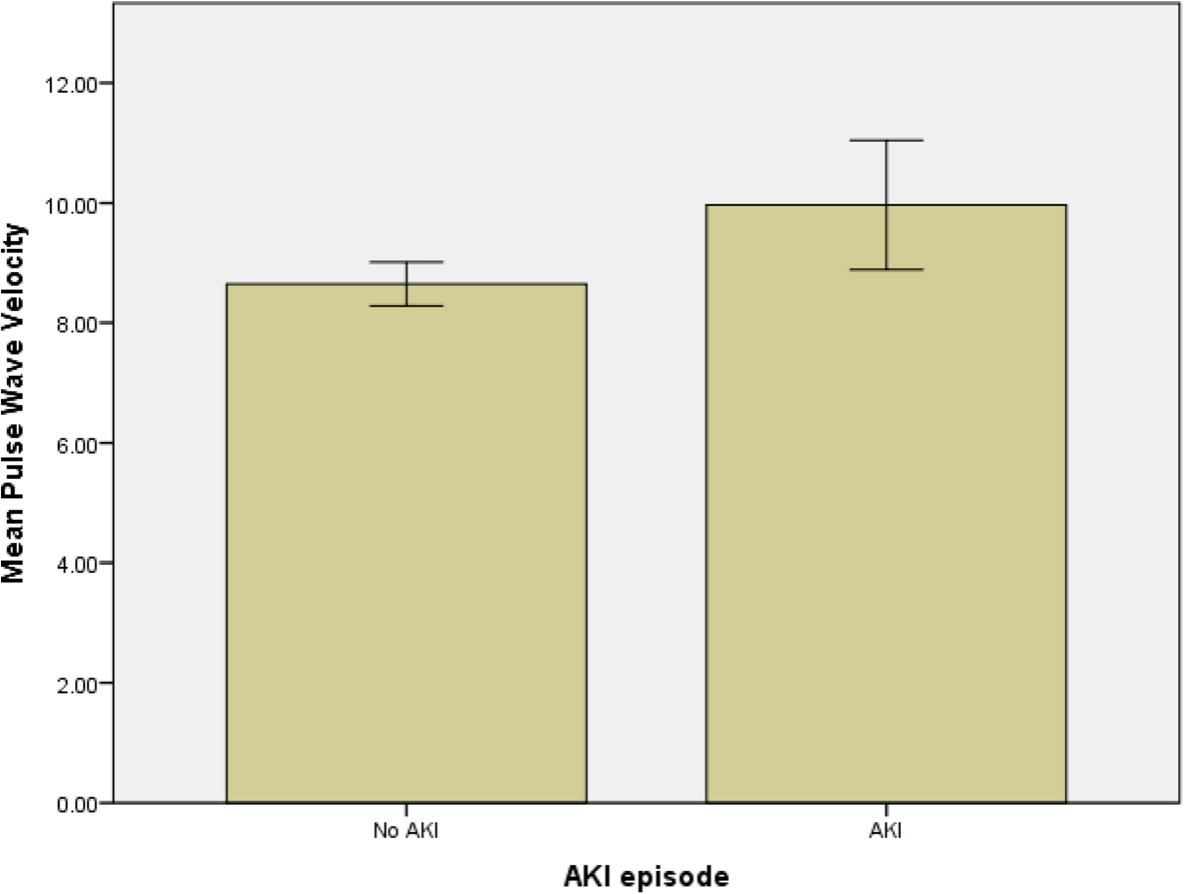
Mean Pulse Wave Velocity values (95% CI) and incidence of cardiac surgery associated Acute Kidney Injury. Mean pulse wave velocity (m/sec); AKI = Acute Kidney Injury

A total of 137 participants were recruited to this study over a 24 month period; demographic data and baseline measurements are shown in Table 1. Participants were predominantly male (85%, *n* = 116) with a mean age of 66.3 years (SD = 9.7 years), and the majority of the participants were of white ethnicity (82%, *n* = 101). None of the participants had a history of pre-operative renal disease or were in an unstable state pre-operatively. Forty patients (29%) developed AKI according to the KDIGO criteria [14]. Table 1 shows a comparison of the variables at the study assessment between those patients who suffered a post-operative AKI event (AKI) and those patients who did not (No AKI). Statistical significance is defined as p_H0_ < 0.05 throughout. Figure 1 displays Pulse Wave Velocity values and incidence of cardiac surgery associated Acute Kidney Injury.

**Table 1:** Summary of descriptive statistics and univariate tests for association with acute kidney injury

Variable	No AKI	AKI	p
Age (years)	67.0 (61.0–73.7)^b^	71.5 (56.7–74.7)^b^	0.405^*^
Systolic blood pressure (mm Hg)	133.8 (17.3)^a^	136.0 (10.9)^a^	0.578^$^
Heart rate (bps)	65.0 (56.0–72.0)^b^	66.5 (59.0–76.2)^b^	0.645^*^
Pulse wave velocity (ms^−1^)	8.3 (7.3–9.9)^b^	9.3 (8.4–10.7)^b^	0.049^*^
eGFR (ml^−1^.min^− 1^.1.73 m^−2^)	78.5 (68.2–90.0)^b^	53.0 (42–65.2)^b^	< 0.001^*^
Diabetes (%yes)	21.4%	50.0%	0.007^#^
Ejection fraction category (%good)	69.2%	65.0%	0.912^#^
Gender (%male)	87.2%	70.0%	0.049^#^

(Descriptive statistics: ^a^ denotes mean (sd); ^b^ denotes median (IQR). Statistical tests: * denotes independent samples median test; $ denotes independent groups t-test; # denotes chi-squared test)

Patients who suffered an AKI event had lower values of eGFR at baseline (*p* < 0.001), a higher probability of having diabetes, (*p* = 0.007), and a greater Pulse Wave Velocity (*p* = 0.049) compared to those in the no AKI group. A higher percentage of women had AKI than men.

It is recognised that multiple tests will result in inflation of overall Type I error but these results are being used as preliminary indicators of possible predictors in the modelling process. All variables were initially included in the models.

Logistic regression utilising a backwards step-regression model as an exploratory analysis, followed by confirmatory hierarchical modelling using forced entry was used to determine which factors independently predicted a post-operative AKI event after checking for the validity of the analytical assumptions. Factors used in the hierarchical models were added in the following order, determined by the strength of the association with AKI in univariate tests (strongest association first):

- eGFR
- Diabetes
- Pulse Wave Velocity
- Gender
- Age
- Systolic blood pressure
- Heart rate
- Ejection fraction category

It is recommended that the number of predictor variables for logistic regression modelling is limited to one predictor for every 10 events, where an event is defined as the outcome of interest (in this case, an occurrence of AKI) [15]. This has been modified more recently to (a cautious) 5–9 events per variable [16]. There were 20 reported cases of AKI (the outcome measure) in the data and therefore a maximum of 4 predictors are recommended for this analysis, as follows;-.

- eGFR
- pulse wave velocity
- age
- gender

This is in agreement with the exploratory backwards stepwise model. A final, forced entry model was constructed with these predictor variables. This final model has sufficient numbers of events for stability with four predictors. eGFR, age, gender, PWV and participants receiving diabetic management were all statistically significant predictors of post-operative AKI and. as shown in Table 2.

**Table 2:** Final model parameters

	B	S.E.	Wald	p	Exp(B)	95% C.I.for EXP(B)	
						Lower	Upper
eGFR	−.157	.035	20.211	<.001	.855	.798	.915
PWV	.431	.158	7.453	.006	1.538	1.129	2.096
Age	−.140	.052	7.271	.007	.870	.786	.963
Gender	−1.912	.896	4.555	.033	.148	.026	.855
Constant	15.396	4.815	10.226	.001	4,856,062.071		

## Discussion

This is the first study to investigate the relationship between PWV and development of AKI post elective CABG surgery; there is, however, previously published data that supports an association between increased levels of AS and reduced kidney function [9]. The results of this current exploratory study demonstrate that higher levels of kidney function (as measured by eGFR), being male, and being younger were associated with a lower risk of participants developing post-operative AKI. These results are consistent with previous studies [7] demonstrating preoperative renal impairment (eGFR < 60 ml/min, creatinine > 2.1 mg/dl) and female sex as independent risk factors for Cardiac Surgery associated AKI (CSA-AKI). Our study also revealed a significant association between pre-operative arterial stiffness (as measured by PWV) and the risk of developing AKI after elective CABG surgery. There was an increased odds ratio of developing AKI of 1.5 with every unit increase in PWV. Earlier work by Kidher et al. [17] reported a non-significant weak correlation between PWV and post aortic valve replacement surgery-AKI in patients with normal to mild levels of pre-operative renal impairment. This current study, which benefited from a larger sample of participants with normal to mild pre-operative renal impairment, appears to contradict this earlier work that didn’t show a significant association between PWV and CSA-associated AKI although we do recognise that the cardiac surgery between the two studies is different.

The mechanism of injury in CKD from increased AS is thought to be related to barotrauma inflicted on glomeruli in a “stiff” vascular system [9]. Preventive strategies for CSA-AKI are limited and the evidence for most interventional therapies is not substantive. The use of a simple non-invasive AS measurement technique, such as PWV, was shown to offer a quick and reliable way to predict those patients at risk of CSA-AKI in our exploratory study. As arterial stiffness is potentially modifiable with therapeutic strategies, reducing AS in potential candidates for cardiac surgery at high risk for AKI, may reduce the risk of CSA-AKI. By identifying those patients at risk of AKI, there can also be increased awareness and vigilance pre- and post-cardiac surgery.

Several limitations need to be acknowledged. Firstly, our study consists of a predominantly caucasian population undergoing elective non-valve repair CABG surgery so there is a need be cautious when extrapolating these results to other patient populations. Secondly, the small number of post-operative AKI episodes meant that we had to restrict the number of dependent variables in the multivariate model. Measurement of PWV as a predictor of CSA-AKI in a larger patient population would be of interest.

## Conclusion

The results from this exploratory study indicate that PWV, assessed prior to elective non-valve repair CABG surgery, independently predicts post-operative AKI events. PWV is a simple, non-invasive technique that could potentially be used to help in stratifying the risk of post-operative AKI following cardiac surgery. Importantly, unlike other risk factors such as eGFR, there is potential to modify AS pre-cardiac surgery and reduce the risk of CSA-AKI. Further research to evaluate potential therapeutic strategies to reduce AS is warrented.

## Abbreviations

AKI: Acute kidney injury
AKIN: Acute Kidney Injury Network
AS: Arterial stiffness
BP: Blood pressure
CABG: Coronary artery bypass graft
CKD: Chronic kidney disease
CSA-AKI: Cardiac surgery-associated acute kidney injury
eGFR: Estimated glomerular filtration rate
ESRF: End stage renal failure
HR: Heart rate
IDMS: Isotope dilution mass spectrometry
KCH: King’s College Hospital
KDIGO: Kidney Disease Improving Global Outcomes
LVH: Left ventricular hypertrophy
NCEPOD: National Confidential Enquiry into Patient Outcomes and Death
NIHR/HEE: National Institute for Health Research/Health Education England
PWV: Pulse Wave Velocity
RIFLE: Risk, Injury, Failure; Loss, End-Stage Renal Disease
